# Innovative Alternatives for Continuous *In Vitro* Culture of *Babesia bigemina* in Medium Free of Components of Animal Origin

**DOI:** 10.3390/pathogens9050343

**Published:** 2020-05-01

**Authors:** Jesús A. Álvarez Martínez, Julio V. Figueroa Millán, Massaro W. Ueti, Carmen Rojas-Martínez

**Affiliations:** 1 Babesia Unit-CENID-Salud Animal e Inocuidad, INIFAP, Carr. Fed. Cuernavaca-Cuautla No. 8534, Col. Progreso, Jiutepec, Morelos C.P. 62550, Mexico; alvarez.jesus@inifap.gob.mx (J.A.Á.M.); figueroa.julio@inifap.gob.mx (J.V.F.M.); 2 Animal Disease Research Unit, Agricultural Research Service, US Department of Agriculture, Pullman, WA 99164-7040, USA

**Keywords:** *Babesia bigemina*, *in vitro* culture, animal component-free medium, CD-lipids

## Abstract

In this study, we report *Babesia bigemina* proliferation in culture medium free of components of animal origin supplemented with a lipid mixture. *Babesia bigemina* continuously proliferated in VP-SFM with a higher percent parasitized erythrocyte as compare to using other animal component-free culture media. Compared with Advanced DMEM/F12 (ADMEM/F12), VP-SFM had a similar percent parasitized erythrocyte (PPE). Supplementation of VP-SF with a lipid acid mixture improved *B. bigemina* proliferation *in vitro* culture, with a maximum PPE of 11.3%. Growth of *B. bigemina* in a perfusion bioreactor using VP-SFM medium supplemented with lipid mixture resulted in a PPE above 28%. In conclusion, we demonstrated that *B. bigemina* proliferated in an animal component-free medium supplemented with the fatty acid mixture. This innovation to *B. bigemina* *in vitro* culture method presented herein is an important source of biological material for live vaccine production and understanding the mechanisms and molecules involved in parasite attachment and invasion of bovine erythrocytes.

## 1. Introduction

Bovine babesiosis is a tick-borne disease caused by *Babesia bigemina* and *Babesia bovis*. This disease affects bovines in tropical and subtropical areas and creates a negative economic impact on the cattle industry [1]. Severe adverse effects occur during clinical disease, including weight loss, decrease in meat and milk production, and, in some cases, death [2,3]. *Rhipicephalus microplus* and *R. annulatus* are the vectors for *B. bigemina* and *B. bovis*. There is no biological animal model to study these parasites other than bovines. The success of *Babesia in vitro* growth in erythrocytes using the microaerophilic stationary phase system (MASP) facilitated the study of gene regulation necessary for parasite development, drug efficiency evaluation, and *Babesia* parasite attenuation for vaccines [4,5,6,7]. Also, due to the advance of engineering technology, the *in vitro* culture of *B. bigemina* and *B. bovis* allowed transfection of these parasites, which has aided the understanding of parasite biology [8,9]. Importantly, more than 100 species of *Babesia* have been isolated. However, few *Babesia* species have been successfully cultivated *in vitro* system [10]. The primary concern is that to maintain continuous *in vitro Babesia* growth is the requirement of high sera concentrations in the growth medium.

Like other *Babesia* spp., *B. bigemina* was cultured in media that contained animal-derived components including 40% of bovine sera. This parasite was first cultivated *in vitro* more than 30 years ago [11]. A recent report in our laboratory using Dulbecco’s modified Eagle medium/F-12 (Advanced DMEM/F12) that contains components of animal origin, supplemented with insulin-transferrin-selenite and putrescine showed that *B. bigemina* successfully proliferated in culture media without bovine sera supplementation [12]. Culture media without components of animal origin or supplementation with animal products to propagate *Babesia* spp. are required to standardize *in vitro* culture methods among laboratories. Reports showed that culture media formulated without animal products are available and used to proliferate protozoan parasites and virus under *in vitro* conditions. A study showed that culture media without animal products successfully maintained the growth of *Toxoplasma gondii* in *in vitro* culture [13]. Another study showed culture media-free of the animal product was used to grow mammalian cell lines for virus production [14,15,16,17,18]. A chemically defined mixture of lipids as a supplement added in culture media-free of animal products resulted in superior growth of CHO cells to produce recombinant proteins [19]. Similarly, lipids added to culture medium increased lentiviral vector productivity and infectivity of the HEK 293 cell line [20]. 

In this study, we investigated whether culture media, free of animal components, supplemented with a lipid mixture would support the growth of *B. bigemina*. We determined that *B. bigemina* proliferated in VP-SFM medium supplemented with a lipid mixture. In contrast, the other three animal component-free culture media supplemented with or without lipid mixture failed to maintain *B. bigemina* growth. Using a perfusion bioreactor system, VP-SFM medium supplemented with lipid mixture improved the parasitemia to over 29% of parasitized erythrocytes. These larger numbers of parasites harvested from *in vitro* culture can be an important source of biological material for the development of strategies to control *B. bigemina*.

## 2. Materials and Methods

### 2.1. Ethical Statement

A five-year-old *Bos taurus* (Holstein Friesian) bovine was the source of erythrocytes to maintain *in vitro B. bigemina* growth. This bovine was certified free of *Babesia* spp., *Anaplasma marginale*, brucellosis, tuberculosis, leucosis, IBR, and BDV. Animal care and use protocol number were NOM-062-ZOO-1999 “Technical specifications for production, care and use of laboratory animals” (http://www.senasica.gob.mx/?doc¼743). 

### 2.2. Bovine Erythrocytes

Whole blood was collected from the jugular by venipuncture into a flask containing glass beads [21]. Defibrinated blood was centrifuged at 450× *g*, 4 °C for 30 min. Serum was removed, and the buffy coat discarded. The erythrocytes were washed five times as follows: the pellet of erythrocyte was suspended 1/2 with VYM solution + antioxidant mixture (v/v) and centrifuged at 450× *g*, 4 °C for 30 min. Erythrocytes were suspended in the VYM solution at a PCV of 30% and stored at 4 °C [12].

### 2.3. Culture Media

Four culture media free of animal components were used in this study, including VP-SFM (Gibco^®^), CD-CHO (Gibco^®^), CD-Hydrolyzed (Sigma-Aldrich, St. Louis, MO, USA), and CD-CHO (Sigma-Aldrich St. Louis, MO, USA). The Advanced-DMEM/F12 (Gibco^®^) that contains animal components was used as the control for *B. bigemina* growth (Table 1). 

All culture media were buffered with 25 mM 2-[(2-hydroxy-1,1-bis(hydroxymethyl)ethyl)amino] ethane sulfonic, *N*-[Tris(hydroxymethyl)methyl]-2-aminoethanosulphonic (TES) (Sigma-Aldrich, St. Louis, MO, USA). An antioxidant mixture was added to 2 mM of L-glutamine (Sigma-Aldrich, St. Louis, MO, USA) (GIBCO^®^). The pH was adjusted (6.8) for all culture media and sterilized by filtration with a 0.22 µm membrane (Millipore). All media were supplemented with different concentrations of CD-Lipid (CORNING^®^) (Table 2).

### 2.4. Parasite and In Vitro Culture

The *B. bigemina* obtained and proliferated *in vitro* was used in this study [12]. Also, *B. bigemina* was adapted to continuous proliferation in ADMEM/F12. *Babesia bigemina* cultures were maintained at 37 °C in a saturated atmosphere of 90% N_2_, 5% CO_2_, and 5% O_2_ [22]. Fresh culture ADMEM/F12 medium was replaced every 24 h to maintain the growth of the parasites [12].

### 2.5. Selection of an Animal Component-Free Culture Medium

The effect of four animal component-free culture media without supplementation was evaluated on the proliferation of *B. bigemina*. The percentage of parasitized erythrocytes (PPE) was daily determined by Giemsa-stained blood smears. All assays began with a 1% parasitemia, and when PPE reached 4%, subcultures were carried out. Subcultures were adjusted to 1% parasitemia by adding uninfected erythrocytes [23]. 

### 2.6. Effect of Lipids on In Vitro Proliferation of *B. bigemina*

The effect of a chemically defined lipid mixture on growth of *B. bigemina* was also evaluated. VP-SFM medium was supplemented with five different concentrations of a commercial lipid mixture to determine the optimum concentration of lipid mixture for the growth of *B. bigemina* (Table 2). Dilutions of the lipids were performed in VP-SFM medium.

### 2.7. In Vitro Proliferation of *B. bigemina* in a Perfusion Bioreactor

*Babesia bigemina* was cultured with a VP-SFM medium supplemented with lipid mixture in a 75 cm^2^ culture flask. As a control, *B. bigemina* was cultured in another culture flask with ADMEM/F12. Briefly, harvested parasites from each flask were applied into a perfusion bioreactor system (FiberCell^®^ System, New Market, MD, USA) as previously described [12,23] for large-scale production of parasites. The bioreactors were kept at 37 °C under a saturated atmosphere of 90% N_2_, 5% CO_2_, and 5% O_2_. The initial parasitemia was 5% in a package of 10 mL erythrocytes. Then, 40 mL of uninfected erythrocytes was added and mixed before introducing them to the bioreactor cartridge. The culture medium was supported by a peristaltic pump to continuously circulate through the system, and the medium was replaced every 24 h with fresh medium. Parasite proliferation was verified at 24 h intervals and subcultures performed every 24–48 h.

### 2.8. Statistical Analysis

The data were analyzed as differences of percent parasitized erythrocytes for the *B. bigemina in vitro* cultures using one-way ANOVA followed by a Dunnett’s test to compare means from different experimental groups against a control group. A Student’s *t*-test was used to assess continuous culture with VP-SFM compared to ADMEM/F12 and to compare VP-SFM supplemented with lipid mixture vs. ADMEM/F12. 

## 3. Results

### 3.1. Selection of an Animal Component-Free Culture Medium

**Figure 1:**
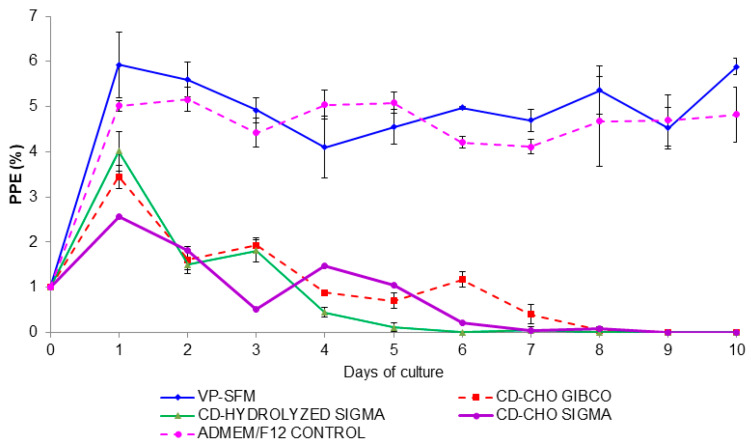
*Babesia bigemina* growth curve using animal component-free media. The percent of parasitized erythrocyte (PPE) was adjusted to 1% when PPE reached 4%. If the PPE was ≤1 then a 1:2 subculture was carried out. Subcultures are not shown.

The VP-SFM medium performed the best for *B. bigemina* proliferation as compared to other culture media free of animal components. There was a significant difference based on the PPE values between VP-SFM and other media (*p* < 0.05). Overall, the maximum PPE values observed for VP-SFM was 6.0%, CD-CHO (Gibco) was 3.44%, CD-Hydrolyzed (Sigma) was 4.01%, CD-CHO (Sigma) was 2.57% and the control medium, ADMEM/F12, was 5.0% (Figure 1). 

**Figure 2:**
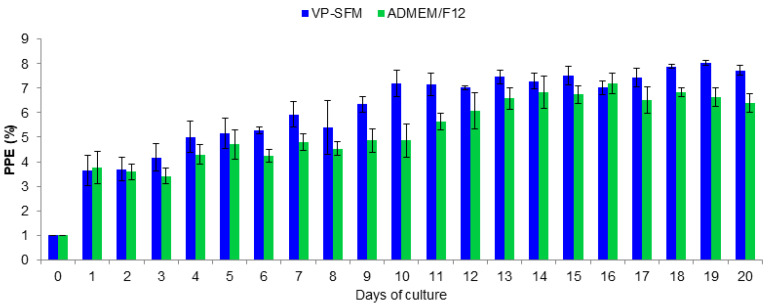
Comparison of *Babesia bigemina* proliferation between VP-SFM and ADMEM/F12 control medium. The percentage of parasitized erythrocytes (PPE) was adjusted to 1% when reached 4%. If the PPE was ≤1 then a 1:2 subculture was carried out. Subcultures are not shown.

The growth of *B. bigemina* was similar in culture with VP-SFM or ADMEM/F12. At 14 days of cultures, the PPE values remained greater than 4% for VP-SFM or ADMEM/F12. In contrast, the PPE values using CD-CHO, CD-hydrolyzed, and CD-CHO as media started declining on day two. In these media, parasites were not detected after day nine. Comparing proliferation of *B. bigemina* between VP-SFM and ADMEM/F12 revealed that the maximum PPE mean values were 8.0% and 7.2%, respectively (Figure 2). 

Based on the percentage of parasitized erythrocytes (PPE), there was no significant difference between these two media (*p* > 0.05). The continuous proliferation of *B. bovis* using the VP-SFM culture medium was successfully performed during more than 350 subcultures carried out every 24–48 h (data not shown).

### 3.2. Effect of the Supplementation with Chemically Defined Lipid on Proliferation of *B. bigemina*

The proliferation of *B. bigemina* was improved by adding a chemically defined lipid mixture. Supplementation of VP-SFM medium with a lipid mixture at a concentration of 0.1 mg/L of myristic acid, palmitic acid, palmitoleic acid, stearic acid, oleic acid, linoleic acid, acid stearic, 0.02 mg/L of arachidonic acid, and 2.2 mg/L of cholesterol resulted in improvement of *B. bigemina* growth *in vitro* culture. There was a significant difference between treatments (*p* < 0.05). In this lipid concentration, the percentage of parasitemia increased from 1.0% to the highest mean value of 11.35%. Other concentrations of lipid mixture treatments showed parasitemia values below 9% (Table 3). 

**Figure 3:**
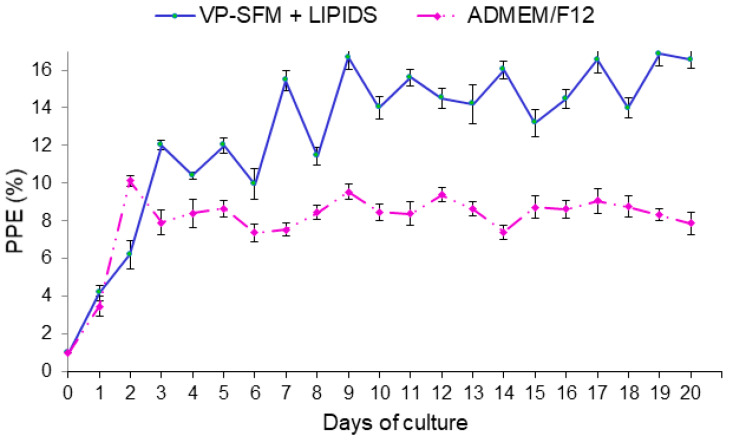
Comparison of growth curve of *Babesia bigemina* proliferation using VP-SFM + lipid with ADMEM/F12 control medium. The parasitemia was adjusted to 1% when the percentage of parasitized erythrocytes (PPE) reached 4. If the PPE was ≤1 then a 1:2 subculture was carried out. Subcultures are not shown.

Continuous *B. bigemina* culture for 20 cycles in lipid supplemented VP-SFM improved the parasitemia values above 12%. At day 19, the maximum PPE was 16.85%. In contrast, continuous *B. bigemina* culture using ADEM/F12 showed PPE values below 10% (Figure 3). Based on parasitemia values, there was a significant difference between treatments (*p* < 0.05).

### 3.3. In Vitro Proliferation of B. bigemina in a Perfusion Bioreactor System

**Figure 4:**
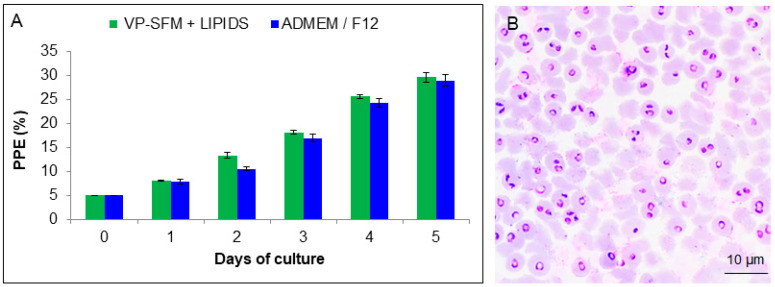
*Babesia bigemina* proliferation in a perfusion bioreactor. (**A**) Growth comparison of *B. bigemina* between VP-SFM + lipid mixture and ADMEM/F12 control medium. (**B**) Giemsa-stained blood smear showing *B. bigemina* parasitized erythrocytes.

*In vitro* proliferation of *B. bigemina* using a perfusion bioreactor system showed a parasitemia of 29.63% using the new lipid supplemented VP-SFM culture medium. *Babesia bigemina* growth using ADMEM/F12 had a parasitemia of 28.95%, (Figure 4A). There was no significant difference between the two treatments (*p* > 0.05). Detectable morphological changes were not observed in smears by light microscopy (Figure 4B).

## 4. Discussion

This is the first report of the continuous *in vitro* growth of *B. bigemina* in a medium free of components of animal origin supplemented with a lipid mixture. Among four different animal component-free culture media, *B. bigemina* proliferated continuously in VP-SFM medium with and without lipid mixture supplementation. 

The previous study in our laboratory demonstrated that *B. bigemina* successfully proliferated in ADMEM/F12 serum-free supplemented with insulin-transferrin-selenite and putrescine. Importantly, putrescine may be critical to achieving the growth of *B. bigemina* in a serum-free medium. Putrescine is a polyamine previously reported as essential for the proliferation of *B. bigemina* and *B. bovis* [12,23] and necessary for other protozoa such *P. falciparum* [24], *Toxoplasma*, and *Leishmania*. These parasites can regulate endogenous levels of putrescine [25]. However, *in silico* analysis of the *B. bovis* genome sequence revealed that this parasite does not have the ability for de novo polyamine biosynthesis [26]. It has been suggested that parasites acquire polyamines from the external environment. Other studies have demonstrated that polyamines such as putrescine, spermidine, and spermine facilitate the rapid proliferation of *P. falciparum* within erythrocytes [27].

In this study, we demonstrated that a relevant factor of the achievement by the use of VP-SFM probably is because one of its components is putrescine for the growth of *B. bigemina in vitro* culture using VP-SFM medium, a medium free of components of animal origin. Also, the supplementation of VP-SFM with a lipid mixture improved *B. bigemina* growth. There is no report showing a combination of animal component-free culture medium and lipids for the proliferation of *Babesia* spp., or other protozoan parasites. The lipid mixture is a source of energy, structural components for cell membranes, transport, and signaling systems [28]. A previous report suggested that lipid metabolism is vital in pathogen replication [29], as well as necessary for *P. falciparum* merozoite stage survival during the intraerythrocytic phase of infection [30]. It has been proposed that exogenous incorporation of fatty acids for the growth and maturation of the parasite in the trophozoite stage is required [31,32]. *Plasmodium falciparum* can modify and control the phospholipids composition of the erythrocyte membrane [33]. *Plasmodium falciparum* increases intracellular levels of fatty acids, such as oleic acid. However, the mechanism by which parasites acquire the fatty acids is unknown [34]. Additionally, *P. falciparum* is capable of synthesizing small quantities of de novo fatty acids, and those that are required are obtained from the external environment [26,30]. Another species, *Plasmodium knowlesi* requires large amounts of phospholipids for its development. Nevertheless, these parasites are not able to produce those lipids de novo. Thus, the sources of those lipids are from the erythrocyte membrane or the external medium [35]. 

We showed that VP-SFM combined with a lipid mixture achieved high efficiency of *B. bigemina* proliferation in a perfusion bioreactor reaching a maximum PPE of 29.63%. These values were essentially equal to a previous study using ADEM/F12 medium containing components of animal origin supplemented with insulin-transferrin-selenite and putrescine [12].

## 5. Conclusions

In this study, we demonstrated that VP-SFM medium, free of animal components, supplemented with a lipid mixture supported *B. bigemina* proliferation in an *in vitro* culture system. The absence of animal components in the culture medium is a novel method to maintain *B. bigemina* growth under *in vitro* conditions. This method will allow harvesting of a high number of *B. bigemina* infected erythrocytes that can be an excellent source of parasites for vaccine development or understanding the mechanisms and molecules involved in parasite attachment and invasion of erythrocytes. Future studies are needed to evaluate the *B. bigemina* genome and transcriptome of these parasites adapted to the new animal component-free medium supplemented with a lipid mixture.

## Figures and Tables

**Table 1:** Growth of *B. bigemina* in media free of animal products.

Medium	Serum-Free	Animal-Free Components	Protein-Free
ADMEM/F12	Yes	No	No
CD-CHO (Gibco^®^),	Yes	Yes	Yes
CD-CHO (Sigma-Aldrich)	Yes	Yes	Yes
CD-Hydrolyzed (Sigma-Aldrich)	Yes	Yes	Yes
VP-SFM (Gibco^®^)	Yes	Yes	No

**Table 2:** Lipid concentration used for *in vitro B. bigemina* proliferation.

Treatment	Lipids Mixture Concentration (mg/mL)								
	Myristic	Palmitic	Palmitoleic	Stearic	Oleic	Linolenic	Stearic	Arachidonic	Cholesterol
I	0.2	0.2	0.2	0.2	0.2	0.2	0.2	0.04	4.4
II	0.1	0.1	0.1	0.1	0.1	0.1	0.1	0.02	2.2
III	0.05	0.05	0.05	0.05	0.05	0.05	0.05	0.01	1.1
IV	0.025	0.025	0.025	0.025	0.025	0.025	0.025	0.005	0.55
V	0.0125	0.0125	0.0125	0.0125	0.0125	0.0125	0.0125	0.0025	0.275

Lipid concentration: half-dilution factor.

**Table 3:** Effect of lipid concentration on *in vitro B. bigemina* proliferation.

Treatment Lipid	Days of Culture						
	0	1	2	3	4	5	6
I	1.0%	2.43%	5.71%	8.00%	7.92%	8.96%	8.03%
II	1.0%	4.93%	7.19% *	9.85% *	10.33% *	10.43% *	11.35% *
II	1.0%	4.77%	5.03%	7.91%	8.70%	8.60%	9.04%
IV	1.0%	4.20%	5.08%	7.06%	7.77%	5.25%	8.10%
V	1.0%	3.12%	3.74%	5.49%	5.61%	8.60%	7.06%
VP-SFM	1.0%	1.77%	4.40%	4.99%	6.07%	7.42%	7.43%

Treatment lipid: one-half dilution factor as described in Table 2. Asterisks indicate significant difference between treatments.
